# 1-Octylindoline-2,3-dione

**DOI:** 10.1107/S1600536813031383

**Published:** 2013-11-23

**Authors:** Fatima-Zahrae Qachchachi, Youssef Kandri Rodi, El Mokhtar Essassi, Werner Kunz, Lahcen El Ammari

**Affiliations:** a Laboratoire de Chimie Organique Appliquée, Université Sidi Mohamed Ben Abdallah, Faculté des Sciences et Techniques, Route d’Immouzzer, BP 2202 Fès, Morocco; b Laboratoire de Chimie Organique Hétérocyclique, URAC 21, Pôle de Compétences Pharmacochimie, Université Mohammed V-Agdal, BP 1014 Avenue Ibn Batouta, Rabat, Morocco; c Institute of Physical and Theoretical Chemistry, University of Regensburg, D-93040 Regensburg, Germany; d Laboratoire de Chimie du Solide Appliquée, Faculté des Sciences, Université Mohammed V-Agdal, Avenue Ibn Battouta, BP 1014, Rabat, Morocco

## Abstract

In the title compound, C_16_H_21_NO_2_, the indoline ring and the two ketone O atoms are approximately coplanar, the largest deviation from the mean plane being 0.063 (2) Å. The mean plane through the fused ring system is nearly perpendicular to the mean plane passing through the 1-octyl chain [dihedral angle = 77.53 (17)°]. In the crystal, mol­ecules are linked by C—H⋯O hydrogen bonds, forming a three-dimensional network.

## Related literature

For the biological activity of indoline derivatives, see: Bhrigu *et al.* (2010 ▶); Malhotra *et al.* (2011 ▶); Da Silva *et al.* (2001 ▶); Ramachandran (2011 ▶); Smitha *et al.* (2008 ▶). For the structure of a related compound, see: Mamari *et al.* (2010 ▶).
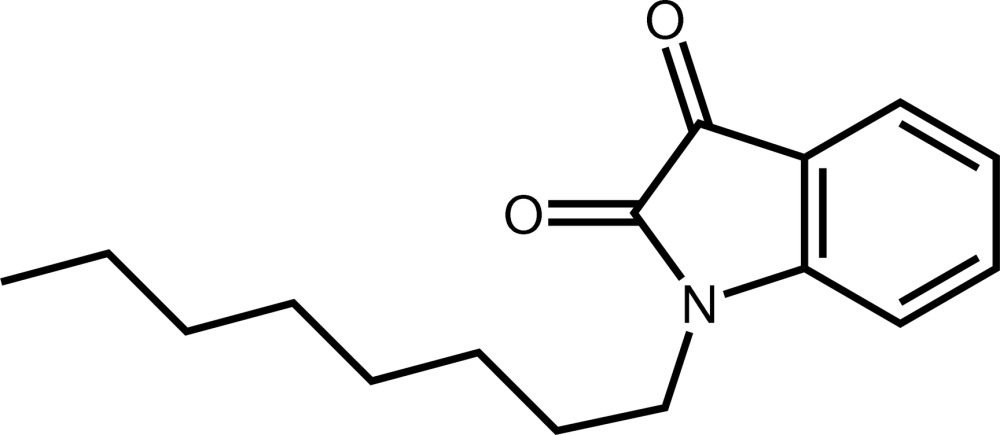


## Experimental

### 

#### Crystal data

C_16_H_21_NO_2_
*M* *_r_* = 259.34
Monoclinic, 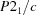

*a* = 20.266 (4) Å
*b* = 4.6925 (1) Å
*c* = 15.7807 (11) Å
β = 108.941 (18)°
*V* = 1419.5 (3) Å^3^
*Z* = 4
Cu *K*α radiation
μ = 0.63 mm^−1^
*T* = 123 K
0.31 × 0.07 × 0.04 mm

#### Data collection

Oxford Diffraction SuperNova (single source at offset, Atlas) diffractometer
Absorption correction: analytical [*CrysAlis PRO* (Oxford Diffraction, 2012 ▶); analytical numeric absorption correction using a multi-faceted crystal model (Clark & Reid, 1995 ▶)] *T*_min_ = 0.899, *T*_max_ = 0.979
13541 measured reflections
2811 independent reflections
2462 reflections with *I* > 2σ(*I*)
*R*_int_ = 0.029

#### Refinement

*R*[*F* ^2^ > 2σ(*F* ^2^)] = 0.057
*wR*(*F* ^2^) = 0.167
*S* = 1.18
2811 reflections
172 parameters
H-atom parameters constrained
Δρ_max_ = 0.49 e Å^−3^
Δρ_min_ = −0.22 e Å^−3^

### 

Data collection: *CrysAlis PRO* (Oxford Diffraction, 2012 ▶); cell refinement: *CrysAlis PRO*; data reduction: *CrysAlis PRO*; program(s) used to solve structure: *SHELXS97* (Sheldrick, 2008 ▶); program(s) used to refine structure: *SHELXL97* (Sheldrick, 2008 ▶); molecular graphics: *WinGX* (Farrugia, 2012 ▶); software used to prepare material for publication: *WinGX* (Farrugia, 2012 ▶) and *publCIF* (Westrip, 2010 ▶).

## Supplementary Material

Crystal structure: contains datablock(s) I. DOI: 10.1107/S1600536813031383/rz5095sup1.cif

Structure factors: contains datablock(s) I. DOI: 10.1107/S1600536813031383/rz5095Isup2.hkl

Supplementary material file. DOI: 10.1107/S1600536813031383/rz5095Isup3.cml

Additional supplementary materials:  crystallographic information; 3D view; checkCIF report

## Figures and Tables

**Table 1:** Hydrogen-bond geometry (Å, °)

*D*—H⋯*A*	*D*—H	H⋯*A*	*D*⋯*A*	*D*—H⋯*A*
C6—H6⋯O1^i^	0.93	2.49	3.156 (3)	129
C6—H6⋯O2^ii^	0.93	2.57	3.260 (3)	131
C4—H4⋯O2^iii^	0.93	2.55	3.470 (3)	170

Symmetry codes: (i) 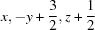; (ii) 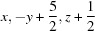; (iii) 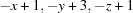.

**Fig. 1.**
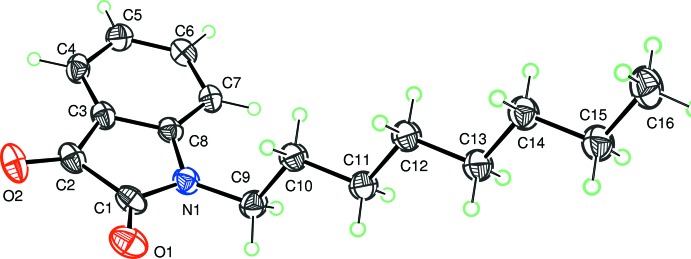
Molecular plot the title compound with displacement ellipsoids drawn at the 50% probability level. H atoms are represented as small circles.

**Fig. 2.**
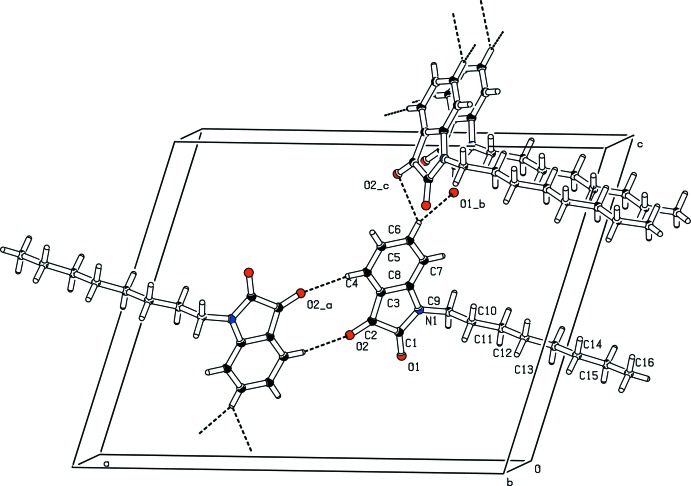
Intermolecular hydrogen interactions (dashed lines) in the title compound. Atoms labelled with suffixes a, b and c are generated by the symmetry operators 1-x, 3-y, 1-z; x, 3/2-y, 1/2+z and x, 5/2-y, 1/2+z, respectively.

## supplementary crystallographic information

### 1. Comment

Isatin, 1*H*-indole-2,3-dione, is a heterocyclic compound of significant importance in medicinal chemistry. It is a synthetically versatile molecule, a precursor for a large number of pharmacologically active compounds. Isatin and its derivatives have aroused great attention in recent years due to their wide variety of biological activities, relevant to application as insecticides and fungicides and in a broad range of drug therapies, including anticancer drugs, antibiotics and antidepressants (Bhrigu *et al.*, 2010; Malhotra *et al.*, 2011; Da Silva *et al.*, 2001; Ramachandran, 2011; Smitha *et al.*, 2008). As a continuation of our research work devoted to the development of isatin derivatives (Mamari *et al.*, 2010), we report in this paper the synthesis of a new indoline-2,3-dione derivative by action of alkyl halides to explore other applications.

The molecule of title compound is build up from a fused five- and six-membered rings linked to a 1-octyl chain and to two ketonic oxygen atoms as shown in Fig. 1. The indoline ring and the two ketonic oxygen atoms are nearly coplanar, with the largest deviation from the mean plane of 0.063 (2) Å for atom O2. The fused ring system plan is nearly perpendicular to the mean plane passing through the 1-octyl chain as indicated by the torsion angle C1–N1–C9–C10 of -93.2 (2)°. In the crystal, the molecules are linked by C–H···O hydrogen bonds (Table 1) to build a three-dimensional network as shown in Fig. 2.

### 2. Experimental

To a solution of isatin (0.5 g, 3.4 mmol) dissolved in DMF (30 ml) was added 1-bromooctane (0.7 ml, 3.4 mmol), potassium carbonate (0.61 g, 4.4 mmol) and a catalytic amount of tetra-n-butylammonium bromide (0.1 g, 0.4 mmol). The mixture was stirred for 48 h and the reaction monitored by thin layer chromatography. The mixture was filtered and the solvent removed under vacuum. The solid obtained was recrystallized from ethanol to afford the title compound as orange crystals (yield: 72%; mp = 317 K).

### 3. Refinement

All H atoms could be located in a difference Fourier map. However, they were placed in calculated positions with C—H = 0.93–0.97 Å and refined as riding on their parent atoms with *U*_iso_(H) = 1.2 *U*_eq_(C) or 1.5 *U*_eq_(C) for methyl H atoms.

### Crystal data

**Table:**

C_16_H_21_NO_2_	*F*(000) = 560
*M**_r_* = 259.34	*D*_x_ = 1.214 Mg m^−^^3^
Monoclinic, *P*2_1_/*c*	Melting point: 317 K
Hall symbol: -P 2ybc	Cu *K*α radiation, λ = 1.5418 Å
*a* = 20.266 (4) Å	Cell parameters from 5321 reflections
*b* = 4.6925 (1) Å	θ = 3.0–73.5°
*c* = 15.7807 (11) Å	µ = 0.63 mm^−^^1^
β = 108.941 (18)°	*T* = 123 K
*V* = 1419.5 (3) Å^3^	Plate, orange
*Z* = 4	0.31 × 0.07 × 0.04 mm

### Data collection

**Table:**

Oxford Diffraction SuperNova (single source at offset, Atlas) diffractometer	2811 independent reflections
Radiation source: SuperNova (Cu) X-ray Source	2462 reflections with *I* > 2σ(*I*)
Mirror monochromator	*R*_int_ = 0.029
Detector resolution: 20.7092 pixels mm^-1^	θ_max_ = 73.7°, θ_min_ = 4.6°
ω scans	*h* = −24→25
Absorption correction: analytical [*CrysAlis PRO* (Oxford Diffraction, 2012); analytical numeric absorption correction using a multi-faceted crystal model (Clark & Reid, 1995)]	*k* = −5→5
*T*_min_ = 0.899, *T*_max_ = 0.979	*l* = −17→19
13541 measured reflections	

### Refinement

**Table:**

Refinement on *F*^2^	Primary atom site location: structure-invariant direct methods
Least-squares matrix: full	Secondary atom site location: difference Fourier map
*R*[*F*^2^ > 2σ(*F*^2^)] = 0.057	Hydrogen site location: inferred from neighbouring sites
*wR*(*F*^2^) = 0.167	H-atom parameters constrained
*S* = 1.18	*w* = 1/[σ^2^(*F*_o_^2^) + (0.0633*P*)^2^ + 1.1901*P*] where *P* = (*F*_o_^2^ + 2*F*_c_^2^)/3
2811 reflections	(Δ/σ)_max_ < 0.001
172 parameters	Δρ_max_ = 0.49 e Å^−^^3^
0 restraints	Δρ_min_ = −0.22 e Å^−^^3^

### Special details

**Table:**

Geometry. All e.s.d.'s (except the e.s.d. in the dihedral angle between two l.s. planes) are estimated using the full covariance matrix. The cell e.s.d.'s are taken into account individually in the estimation of e.s.d.'s in distances, angles and torsion angles; correlations between e.s.d.'s in cell parameters are only used when they are defined by crystal symmetry. An approximate (isotropic) treatment of cell e.s.d.'s is used for estimating e.s.d.'s involving l.s. planes.
Refinement. Refinement of *F*^2^ against all reflections. The weighted *R*-factor *wR* and goodness of fit *S* are based on *F*^2^, conventional *R*-factors *R* are based on *F*, with *F* set to zero for negative *F*^2^. The threshold expression of *F*^2^ > 2σ(*F*^2^) is used only for calculating *R*-factors(gt) *etc*. and is not relevant to the choice of reflections for refinement. *R*-factors based on *F*^2^ are statistically about twice as large as those based on *F*, and *R*- factors based on all data will be even larger.

### Fractional atomic coordinates and isotropic or equivalent isotropic displacement parameters (Å^2^)

**Table:**

	*x*	*y*	*z*	*U*_iso_*/*U*_eq_	
O1	0.33354 (9)	0.7667 (4)	0.34657 (10)	0.0397 (4)	
O2	0.44511 (8)	1.1900 (4)	0.42454 (10)	0.0385 (4)	
N1	0.32574 (9)	0.7836 (4)	0.48939 (11)	0.0272 (4)	
C1	0.35221 (11)	0.8574 (5)	0.42319 (14)	0.0299 (5)	
C2	0.40999 (10)	1.0835 (5)	0.46468 (14)	0.0288 (5)	
C3	0.40798 (10)	1.1295 (4)	0.55543 (13)	0.0258 (4)	
C4	0.44509 (10)	1.3124 (5)	0.62281 (15)	0.0296 (5)	
H4	0.4791	1.4325	0.6148	0.036*	
C5	0.43015 (11)	1.3116 (5)	0.70275 (14)	0.0308 (5)	
H5	0.4540	1.4343	0.7488	0.037*	
C6	0.37992 (11)	1.1290 (5)	0.71428 (13)	0.0290 (5)	
H6	0.3710	1.1310	0.7685	0.035*	
C7	0.34230 (10)	0.9420 (5)	0.64691 (13)	0.0272 (4)	
H7	0.3089	0.8196	0.6553	0.033*	
C8	0.35685 (9)	0.9471 (4)	0.56786 (13)	0.0241 (4)	
C9	0.26652 (10)	0.5930 (5)	0.47581 (15)	0.0303 (5)	
H9A	0.2732	0.4819	0.5298	0.036*	
H9B	0.2645	0.4618	0.4275	0.036*	
C10	0.19768 (10)	0.7542 (5)	0.45315 (15)	0.0300 (5)	
H10A	0.1940	0.8837	0.4039	0.036*	
H10B	0.1976	0.8674	0.5045	0.036*	
C11	0.13437 (10)	0.5572 (5)	0.42731 (15)	0.0305 (5)	
H11A	0.1382	0.4262	0.4763	0.037*	
H11B	0.1341	0.4455	0.3754	0.037*	
C12	0.06565 (10)	0.7207 (5)	0.40574 (15)	0.0311 (5)	
H12A	0.0651	0.8230	0.4589	0.037*	
H12B	0.0635	0.8605	0.3597	0.037*	
C13	0.00107 (10)	0.5314 (5)	0.37394 (15)	0.0317 (5)	
H13A	0.0014	0.4298	0.3205	0.038*	
H13B	0.0031	0.3912	0.4199	0.038*	
C14	−0.06690 (11)	0.6980 (5)	0.35310 (15)	0.0327 (5)	
H14A	−0.0683	0.8412	0.3082	0.039*	
H14B	−0.0675	0.7962	0.4069	0.039*	
C15	−0.13189 (11)	0.5123 (5)	0.31923 (16)	0.0358 (5)	
H15A	−0.1306	0.3683	0.3638	0.043*	
H15B	−0.1317	0.4154	0.2650	0.043*	
C16	−0.19929 (12)	0.6838 (6)	0.29952 (18)	0.0429 (6)	
H16A	−0.2386	0.5578	0.2788	0.064*	
H16B	−0.2015	0.8234	0.2542	0.064*	
H16C	−0.2001	0.7778	0.3532	0.064*	

### Atomic displacement parameters (Å^2^)

**Table:**

	*U*^11^	*U*^22^	*U*^33^	*U*^12^	*U*^13^	*U*^23^
O1	0.0396 (9)	0.0494 (10)	0.0293 (8)	0.0081 (7)	0.0102 (7)	−0.0051 (7)
O2	0.0303 (8)	0.0559 (11)	0.0335 (8)	0.0040 (7)	0.0160 (7)	0.0132 (7)
N1	0.0222 (8)	0.0314 (9)	0.0271 (9)	0.0000 (7)	0.0067 (7)	−0.0028 (7)
C1	0.0265 (10)	0.0372 (12)	0.0262 (10)	0.0098 (8)	0.0087 (8)	0.0014 (8)
C2	0.0224 (9)	0.0349 (11)	0.0297 (10)	0.0088 (8)	0.0092 (8)	0.0086 (9)
C3	0.0225 (9)	0.0285 (10)	0.0275 (10)	0.0050 (7)	0.0096 (8)	0.0072 (8)
C4	0.0207 (9)	0.0307 (11)	0.0364 (11)	−0.0008 (8)	0.0078 (8)	0.0049 (9)
C5	0.0267 (10)	0.0319 (11)	0.0312 (11)	0.0000 (8)	0.0059 (8)	−0.0013 (8)
C6	0.0275 (10)	0.0353 (11)	0.0243 (10)	0.0047 (8)	0.0084 (8)	0.0026 (8)
C7	0.0240 (9)	0.0306 (11)	0.0286 (10)	0.0001 (8)	0.0107 (8)	0.0031 (8)
C8	0.0188 (9)	0.0257 (10)	0.0263 (9)	0.0040 (7)	0.0053 (7)	0.0011 (8)
C9	0.0250 (10)	0.0284 (11)	0.0350 (11)	−0.0012 (8)	0.0064 (8)	−0.0031 (8)
C10	0.0247 (10)	0.0299 (11)	0.0340 (11)	0.0014 (8)	0.0074 (8)	−0.0014 (8)
C11	0.0238 (10)	0.0311 (11)	0.0345 (11)	0.0004 (8)	0.0063 (8)	−0.0026 (9)
C12	0.0236 (10)	0.0324 (11)	0.0353 (11)	0.0011 (8)	0.0067 (8)	−0.0013 (9)
C13	0.0253 (10)	0.0337 (12)	0.0347 (11)	0.0000 (8)	0.0079 (8)	−0.0023 (9)
C14	0.0256 (11)	0.0365 (12)	0.0344 (11)	0.0004 (9)	0.0075 (9)	−0.0005 (9)
C15	0.0271 (11)	0.0404 (13)	0.0388 (12)	−0.0014 (9)	0.0092 (9)	−0.0039 (10)
C16	0.0244 (11)	0.0546 (15)	0.0462 (14)	−0.0006 (10)	0.0069 (10)	−0.0003 (12)

### Geometric parameters (Å, º)

**Table:**

O1—C1	1.220 (3)	C10—H10A	0.9700
O2—C2	1.204 (3)	C10—H10B	0.9700
N1—C1	1.365 (3)	C11—C12	1.528 (3)
N1—C8	1.419 (3)	C11—H11A	0.9700
N1—C9	1.456 (3)	C11—H11B	0.9700
C1—C2	1.558 (3)	C12—C13	1.526 (3)
C2—C3	1.462 (3)	C12—H12A	0.9700
C3—C4	1.383 (3)	C12—H12B	0.9700
C3—C8	1.406 (3)	C13—C14	1.524 (3)
C4—C5	1.390 (3)	C13—H13A	0.9700
C4—H4	0.9300	C13—H13B	0.9700
C5—C6	1.387 (3)	C14—C15	1.524 (3)
C5—H5	0.9300	C14—H14A	0.9700
C6—C7	1.398 (3)	C14—H14B	0.9700
C6—H6	0.9300	C15—C16	1.528 (3)
C7—C8	1.372 (3)	C15—H15A	0.9700
C7—H7	0.9300	C15—H15B	0.9700
C9—C10	1.524 (3)	C16—H16A	0.9600
C9—H9A	0.9700	C16—H16B	0.9600
C9—H9B	0.9700	C16—H16C	0.9600
C10—C11	1.526 (3)		
			
C1—N1—C8	110.93 (17)	H10A—C10—H10B	107.8
C1—N1—C9	123.59 (18)	C10—C11—C12	112.44 (18)
C8—N1—C9	124.91 (17)	C10—C11—H11A	109.1
O1—C1—N1	126.7 (2)	C12—C11—H11A	109.1
O1—C1—C2	127.1 (2)	C10—C11—H11B	109.1
N1—C1—C2	106.24 (17)	C12—C11—H11B	109.1
O2—C2—C3	131.6 (2)	H11A—C11—H11B	107.8
O2—C2—C1	123.6 (2)	C13—C12—C11	113.79 (18)
C3—C2—C1	104.83 (16)	C13—C12—H12A	108.8
C4—C3—C8	120.78 (18)	C11—C12—H12A	108.8
C4—C3—C2	131.56 (19)	C13—C12—H12B	108.8
C8—C3—C2	107.65 (18)	C11—C12—H12B	108.8
C3—C4—C5	118.16 (19)	H12A—C12—H12B	107.7
C3—C4—H4	120.9	C14—C13—C12	113.04 (18)
C5—C4—H4	120.9	C14—C13—H13A	109.0
C6—C5—C4	120.4 (2)	C12—C13—H13A	109.0
C6—C5—H5	119.8	C14—C13—H13B	109.0
C4—C5—H5	119.8	C12—C13—H13B	109.0
C5—C6—C7	122.01 (19)	H13A—C13—H13B	107.8
C5—C6—H6	119.0	C13—C14—C15	113.60 (19)
C7—C6—H6	119.0	C13—C14—H14A	108.8
C8—C7—C6	117.09 (19)	C15—C14—H14A	108.8
C8—C7—H7	121.5	C13—C14—H14B	108.8
C6—C7—H7	121.5	C15—C14—H14B	108.8
C7—C8—C3	121.54 (19)	H14A—C14—H14B	107.7
C7—C8—N1	128.20 (18)	C14—C15—C16	112.6 (2)
C3—C8—N1	110.25 (17)	C14—C15—H15A	109.1
N1—C9—C10	112.20 (17)	C16—C15—H15A	109.1
N1—C9—H9A	109.2	C14—C15—H15B	109.1
C10—C9—H9A	109.2	C16—C15—H15B	109.1
N1—C9—H9B	109.2	H15A—C15—H15B	107.8
C10—C9—H9B	109.2	C15—C16—H16A	109.5
H9A—C9—H9B	107.9	C15—C16—H16B	109.5
C9—C10—C11	112.85 (17)	H16A—C16—H16B	109.5
C9—C10—H10A	109.0	C15—C16—H16C	109.5
C11—C10—H10A	109.0	H16A—C16—H16C	109.5
C9—C10—H10B	109.0	H16B—C16—H16C	109.5
C11—C10—H10B	109.0		
			
C8—N1—C1—O1	−175.8 (2)	C6—C7—C8—N1	−179.05 (19)
C9—N1—C1—O1	−4.1 (3)	C4—C3—C8—C7	−0.5 (3)
C8—N1—C1—C2	3.3 (2)	C2—C3—C8—C7	−179.61 (18)
C9—N1—C1—C2	174.96 (17)	C4—C3—C8—N1	179.39 (18)
O1—C1—C2—O2	−2.5 (3)	C2—C3—C8—N1	0.3 (2)
N1—C1—C2—O2	178.42 (19)	C1—N1—C8—C7	177.49 (19)
O1—C1—C2—C3	176.1 (2)	C9—N1—C8—C7	5.9 (3)
N1—C1—C2—C3	−3.0 (2)	C1—N1—C8—C3	−2.4 (2)
O2—C2—C3—C4	1.1 (4)	C9—N1—C8—C3	−173.93 (18)
C1—C2—C3—C4	−177.4 (2)	C1—N1—C9—C10	−93.2 (2)
O2—C2—C3—C8	−180.0 (2)	C8—N1—C9—C10	77.3 (2)
C1—C2—C3—C8	1.6 (2)	N1—C9—C10—C11	172.83 (17)
C8—C3—C4—C5	−0.3 (3)	C9—C10—C11—C12	179.35 (18)
C2—C3—C4—C5	178.5 (2)	C10—C11—C12—C13	176.35 (18)
C3—C4—C5—C6	0.8 (3)	C11—C12—C13—C14	179.79 (18)
C4—C5—C6—C7	−0.5 (3)	C12—C13—C14—C15	178.69 (18)
C5—C6—C7—C8	−0.3 (3)	C13—C14—C15—C16	179.56 (19)
C6—C7—C8—C3	0.8 (3)		

### Hydrogen-bond geometry (Å, º)

**Table:**

*D*—H···*A*	*D*—H	H···*A*	*D*···*A*	*D*—H···*A*
C6—H6···O1^i^	0.93	2.49	3.156 (3)	129
C6—H6···O2^ii^	0.93	2.57	3.260 (3)	131
C4—H4···O2^iii^	0.93	2.55	3.470 (3)	170

Symmetry codes: (i) *x*, −*y*+3/2, *z*+1/2; (ii) *x*, −*y*+5/2, *z*+1/2; (iii) −*x*+1, −*y*+3, −*z*+1.
